# Foodborne Diseases in the Global Community^1^

**DOI:** 10.3201/eid1011.040797_11

**Published:** 2004-11

**Authors:** Elaine Scallan, Paul Berger, Dale Morse

**Affiliations:** * Centers for Disease Control and Prevention, Atlanta, Georgia, USA;; † Environmental Protection Agency, Washington, D.C., USA;; ‡ New York State Department of Health, Albany, New York, USA

**Keywords:** foodborne diseases, food safety, food industry, WHO Global Salm-Surv, PulseNet, OzFoodNet, FoodNet

Globalization of the food industry and rise in air travel has presented new challenges for food safety and has increased the need for global surveillance of foodborne disease.

Established in 2000, WHO Global Salm-Surv has strengthened surveillance capabilities in participating countries by acting as a resource for training and expertise in foodborne disease surveillance and response. Regional training courses and other activities—including an external quality assurance system, reference services, and electronic discussions—aim to improve collaboration among microbiologists and epidemiologists and foster collaboration across sectors. Other World Health Organization activities include regional-specific projects and establishment of sentinel sites with the purpose of estimating the impact of foodborne disease. Policies and guidelines have been developed on the containment of antimicrobial resistance, the investigation and control of foodborne outbreaks, and response to urgent issues. Establishing networks within and between countries and with other networks is considered essential in improving surveillance and food safety throughout the world.

PulseNet is an early warning system for outbreaks of foodborne disease. First established as a national network in the United States in 1996, this network of public health laboratories performs a standardized DNA "fingerprinting" method called pulsed-field gel electrophoresis on foodborne bacteria. PulseNet identifies and labels each fingerprint pattern and permits rapid comparison of these patterns through an electronic database to identify related strains across a wide geographic area. PulseNet Asia Pacific began in 2002 with a planning meeting involving 12 countries or areas from the region. In 2004, while challenges remain, improvements continue on a routine basis, as the network strives to enhance human health and improve the safety of foods within the Asia Pacific region.

The goals of the Australian government initiative (OzFoodNet) are to determine the impact and cause of foodborne diseases, provide foodborne disease data for risk assessments and policy, and train people to investigate foodborne illness. OzFoodNet, FoodNet in the United States, and groups in Canada and Ireland have been collaborating on international projects. These international partnerships are vital for improving and standardizing methodologic approaches and gaining a better understanding of the global impact of foodborne disease. International partnerships are also important when investigating international outbreaks. Outbreaks involving multiple countries are becoming more common with the ever-increasing global movement of food and people; improved surveillance can detect outbreaks even if they are dispersed over a broad area. International collaboration can lead to more effective outbreak control and to global improvements in food safety.

